# Lessons for Control of Heroin-Associated Anthrax in Europe from 2009–2010 Outbreak Case Studies, London, UK

**DOI:** 10.3201/eid2007.131764

**Published:** 2014-07

**Authors:** Aula Abbara, Tim Brooks, Graham P. Taylor, Marianne Nolan, Hugo Donaldson, Maribel Manikon, Alison Holmes

**Affiliations:** Imperial College Healthcare National Health Service Trust, London, UK (A. Abbara, G.P. Taylor, M. Nolan, H. Donaldson, M. Manikon, A. Holmes);; Public Health England, Porton Down, UK (T. Brooks)

**Keywords:** anthrax, heroin, soft tissue infection, skin popping, subcutaneous, injection, sepsis, persons who inject drugs, PWID, Europe, London

## Abstract

Outbreaks of serious infections associated with heroin use in persons who inject drugs (PWIDs) occur intermittently and require vigilance and rapid reporting of individual cases. Here, we give a firsthand account of the cases in London during an outbreak of heroin-associated anthrax during 2009–2010 in the United Kingdom. This new manifestation of anthrax has resulted in a clinical manifestation distinct from already recognized forms. During 2012–13, additional cases of heroin-associated anthrax among PWIDs in England and other European countries were reported, suggesting that anthrax-contaminated heroin remains in circulation. Antibacterial drugs used for serious soft tissue infection are effective against anthrax, which may lead to substantial underrecognition of this novel illness. The outbreak in London provides a strong case for ongoing vigilance and the use of serologic testing in diagnosis and serologic surveillance schemes to determine and monitor the prevalence of anthrax exposure in the PWID community.

## Abstract 2

Serologic surveillance should be implemented to identify the prevalence of this disease in persons who inject drugs.

Heroin-associated anthrax resulting from direct injection or injection under the skin, or “skin popping,” among persons who inject drugs (PWIDs) is a distinct form of anthrax seen during a 2009–2010 outbreak in Scotland and England and again during 2012–2013 in northern Europe and Germany. There are an estimated 281,000 heroin users in England and >50,000 in Scotland (*1*), suggesting that the cases recognized and diagnosed during the outbreaks are the tip of the iceberg.

The first 2 cases of heroin-associated anthrax occurred in the Greater Glasgow and Clyde area of Scotland and were reported on December 10, 2009. By July 2010, there were 47 confirmed case-patients in Scotland, of whom 13 (28%) died (*2*). In January 2010, the first cases outside Scotland were described in England and Germany (*3*); the final outbreak total in England was 6, with 4 deaths (*4*). The last case from this outbreak occurred in October 2010, and Health Protection Scotland declared the UK outbreak over on December 23, 2010; no active surveillance was established afterward (*4*,*5*).

In June 2012, 21 months after the last reported case in the United Kingdom, a fatal case was reported in Regensburg, Bavaria, Germany. As of March 2013, 7 more cases had occurred in the United Kingdom, including 5 in England; 4 patients died during this outbreak: 2 in England, 1 in Scotland, and 1 in Wales. Another 6 cases occurred in Germany, Denmark, and France, bringing the total to 14 as of (March 2013) (*6*–*8*), which suggests that contaminated heroin remains in circulation and that vigilance should be maintained. 

The only reported case of heroin-associated anthrax before this outbreak was during 2000 in an injecting drug user in Norway in whom fatal hemorrhagic encephalitis developed. Although anticipated, no outbreak emerged (*9*).

## Bacillus anthracis

*Bacillus anthracis* is a spore-forming, gram–positive zoonosis which causes infection in humans through contact with contaminated animals or animal products (*10*). It occurs naturally in soil and mainly affects hoofed animals including goats, cattle, and sheep that ingest endospores (*11*–*13*). *B. anthracis* endospores are hardy and resistant to drying, heat, ultraviolet light, and many disinfectants, and can lie dormant in soil for many years (*10*). The endospores can be ingested, inhaled, or enter through skin abrasions after which they are phagocytosed by macrophages, where they germinate, resulting in activation and recruitment of other immune cells (*10*,*11*). In some cases, the bacteria are not destroyed and can activate a program of antigen-presenting cells and migrate toward lymph nodes (*11*); replication in the lymphatic system can then lead to septicemia. *B. anthracis* secretes 3 polypeptides called protective antigen, lethal factor, and edema factor, which combine to form exotoxins (*10*,*11*). These toxins have numerous effects on phagocytes, including impairment of maturation, impairment of chemotaxis of different phagocytes, and inhibition of phagocyte function (*11*). Edema toxin (protective antigen and edema factor) inhibits neutrophil function and lethal toxin (protective antigen and lethal factor) stimulates macrophages to release tumor necrosis factor α and interleukin-1α (*10*). It is possible that the host immune response contributes to the virulence of the pathogen, stimulating greater inflammatory cytokine release and recruitment of cells, including neutrophils, which have an essential role in controlling anthrax infection (*11*).

Typically, infection occurs through entry of the spores through the skin, by ingestion of contaminated meat, or by inhalation; contamination via these routes commonly results in cutaneous, gastrointestinal, or pulmonary anthrax, or may manifest as hemorrhagic meningitis (*10*,*12**,**13*). The intravenous route of exposure has added a new complexity to the immunopathogenic picture and has resulted in novel, severe, and highly variable patterns of manifestation which are described in the clinical cases section of this article. Anthrax has been used as a biowarfare select agent, and there is concern regarding potential future use. In 2001, the greater New York City metropolitan area in the United States was the scene of an attack (*14*) which resulted in at least 22 cases of anthrax, leading to 5 deaths. These cases resulted from a mix of inhalational and cutaneous exposures, affecting mainly persons who had contact with contaminated items sent through mail. This was a deliberate bioterrorist attack in which envelopes containing anthrax spores were delivered to victims. Early recognition of the first case resulted in a prompt public health response and epidemiologic investigations in the United States and internationally (*15*).

## International Heroin Production and Transportation

Afghanistan is now the largest exporter of heroin, accounting for 93% of the world’s supply (*16*). The United Kingdom National Anthrax Outbreak Control Team hypothesized that the heroin responsible for the 2009 outbreak had been contaminated at its likely source in Afghanistan or entered the supply chain by introduction of cutting or dissolving agents or through animal hides used for transport (*17*).

Phylogeographic analysis demonstrated that the anthrax strains responsible for the 2009–2010 outbreak in Scotland were closely related to strains from Turkey and not to prior isolates from Scotland or Afghanistan (*18*). Anthrax is endemic in Turkey, and heroin passing through Turkey may have been contaminated while being transported in contaminated goatskins (*18*). Isolates from the 2009–2010 and 2012–2013 outbreaks and the isolate from the case-patient from Norway in 2000 were subjected to comparative molecular typing by using a 31-marker multilocus variable number tandem repeat analysis and a broad single-nucleotide-polymorphism analysis. Results demonstrated that these strains were almost identical (*8*).

## Clinical Cases

Here we give a firsthand account of 3 persons with anthrax associated with subcutaneous or intraarterial injection of heroin who sought care at different hospitals in the National Hospital Service trust. These cases highlight the spectrum of disease, specific management challenges, and the importance of serologic testing.

**Figure 1:**
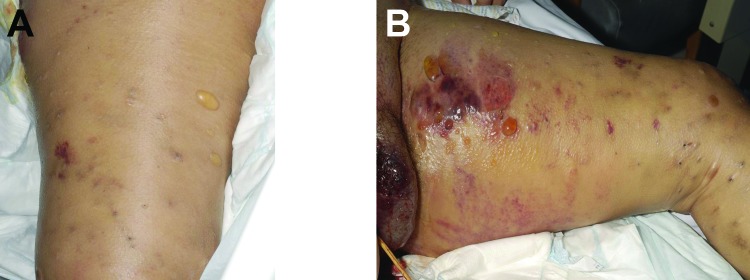
Manifestation of heroin-associated anthrax in patient 1, who injected heroin under the skin of her left thigh. Panel A demonstrates substantial edema and blistering of skin. Manifestation is more pronounced in Panel B, which demonstrates more blistering and bruising.

**Figure 2:**
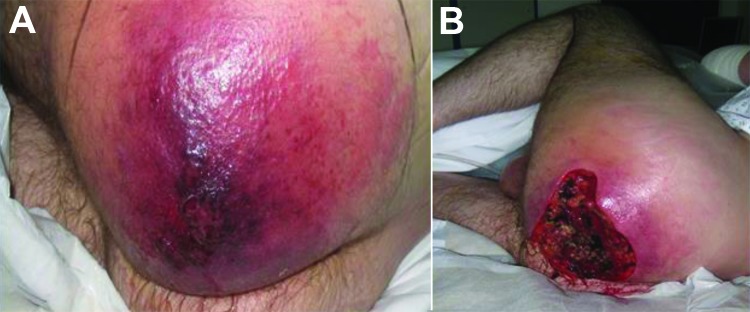
Manifestation of heroin-associated anthrax in patient 1, who injected heroin under the skin of his left buttock 1 week before seeking treatment. Panel A demonstrates severe edema with poorly demarcated erythema. He had debridement within hours of arrival. Panel B demonstrates the extent of debridement required to reach healthy tissue and to reduce the toxin burden; there was copious drainage of fluid and pus as well as bleeding to the area. The tissue between affected and unaffected areas was poorly demarcated.

The manifestations of disease, treatments, and outcomes are summarized in the Table. Patient 1 manifested extensive, painless edema in the left thigh at the subcutaneous injection site. (Figure 1, panels A and B.) Despite remaining lucid and appearing comfortable, she was hemodynamically unstable and initially managed for severe soft tissue infection with septicemia; *B*. *anthracis* was identified from blood cultures the next day. Patient 2 also had extensive tissue involvement at the site of subcutaneous injection to the right buttock; he required extensive tissue debridement (Figure 2, panels A and B). Patient 3 had a pseudoaneurysm of the femoral artery after he injected heroin into that vessel; he was systemically well, although febrile, on arrival to the acute care facility. Samples taken at debridement and cultured were negative for anthrax; however, positive serology indicated recent infection.

**Table:** Details of 3 heroin-associated anthrax patients from the 2009–2010anthraxoutbreak, London, United Kingdom*

Characteristic	Patient 1	Patient 2	Patient 3
Age, y/sex	43/F	30/M	60/M
Comorbidities	HIV, hepatitis C	Hepatitis B, hepatitis C, thromboembolic disease	Hepatitis C, left femoral artery pseudoaneurysm
Route of infection	Subcutaneous injection to left thigh 3 d before admission	Subcutaneous injection to right buttock 1 wk before admission	Injected into left femoral artery
Site affected when patient sought treatment	Extensive involvement: painless edema and blistering of the left thigh, lower abdomen, genitals	Right buttock erythematous, swollen, edematous, and painful; edema extended to genitals	Pulsatile mass at left groin area; no edema or swelling evident
Surgery	Extensive debridement by general surgery and gynecology performed on 2 occasions; skin graft applied later	Early, limited debridement performed on d 1 of hospitalization. Skin graft applied later	On hospital d 1, surgery performed to repair left femoral artery pseudoaneurysm and debridement; further debridement performed at d 19
Anthrax testing results			
Culture	Blood culture of specimen drawn on admission positive in <24 h	Blood and tissue cultured on admission positive 24 h after admission	Blood and tissue cultured on admission negative
Serologic	Positive	Positive	Positive
PCR	Positive	Positive	Negative
Initial antibiotic drugs	Ceftriaxone, clindamycin, vancomycin	Clindamycin, ciprofloxacin, flucloxacillin, vancomycin, gentamicin	Clindamycin, ciprofloxacin, flucloxacillin, benzylpenicillin, metronidazole
Outcome	Initially lucid and comfortable but hemodynamically unstable. Debridement on 2 occasions. Anthrax PCR post–antibiotic drug treatment negative; coagulopathy resolved by day 29 with normal platelets and clotting studies. On day 31, brain stem ischemia developed; died on d 50 after airway complications.	After initial debridement, electively intubated to treat edema causing respiratory compromise. Received AIGIV within 24 h of admission. Vacuum-assisted therapy pump was used, then skin graft, with good outcome. Recovered and was discharged to complete 60 d of ciprofloxacin and clindamycin.	After first surgery on hospital d 1, continued broad-spectrum antibiotic drugs for 10 d. Received a further 14 d of broad-spectrum antibiotic drugs after debridement on d 19. Made a good recovery and was discharged home. Strongly positive serologic results subsequently received.
Test results for blood samples taken at admission (reference range)†			
Leukocyte count (4.2–11.2 x 10^9^ L)	23.1	16.8	10.1
Neutrophils (2.0–7.1 x 10^9^/L)	14.6	14.6	4.9
CRP (0–4 mg/L)	179	71	230
Hemoglobin (13.0–16.8 g/dL)	15.7	6.7	9.8
INR (1.0)	4.4	1.5	1.0
Platelets (130–370 x 10^9^/L)	374	30	238
Creatinine (60–125 μmol/L)	385	488	137
Albumin (30–45 g/L)	24	23	30

*Patients 1, 2, and 3 represent the diversity of the cases seen and the spectrum of manifestation caused by heroin-associated anthrax. Clinical features associated with this condition include the degree of edema present, the absence of the eschar associated with cutaneous anthrax, and the biphasic nature of the illness; in the severe cases, Patients 1 and 2 experienced multiorgan dysfunction and coagulopathy. AIGIV, anthrax immune globulin intravenous; CRP, C-reactive protein; INR, international normalized ratio. †Reference ranges from Imperial College Healthcare (http://www.imperial.nhs.uk/services/pathology/index.htm).

These cases demonstrate the range of clinical manifestations, from relative hemodynamic stability (patient 3) to multiorgan failure requiring intensive care support (patients 1 and 2.) Clinical features of anthrax-associated soft tissue infection that differentiate it from that caused by other bacteria include the degree of edema affecting surrounding tissue (*18*,*19*), the excessive bleeding at the time of surgery, and the lack of a clear demarcation between affected and unaffected tissue.

In patients that have predominantly soft tissue manifestations, there is a notable absence of the eschar that is typical of cutaneous anthrax, marking this manifestation as a distinct form. Despite extensive tissue involvement as seen in patients 1 and 2, the degree of discomfort displayed by the patients was markedly less than would be expected, and they appeared deceptively comfortable despite the clinical features and laboratory tests that indicated severe infection. Both of these patients were coagulopathic with substantial renal failure requiring hemofiltration. The leukocyte count and C-reactive protein level were not as elevated as might be expected, given the degree of tissue involvement and the degree of organ failure evident; this was particularly evident in patient 2.

Patients 1 and 2, as seen in the Scottish anthrax case-patients, exhibited a biphasic illness with an initial recovery, then further deterioration (*20**,**21**,**22*). Radiologic features of pulmonary anthrax include pleural effusions, mediastinal widening, paratracheal or hilar fullness, and parenchymal infiltrates. Patients 1 and 2 showed chest x-ray evidence of severe pulmonary edema shortly after arrival.

Patients 1 and 2 were managed by a multidisciplinary team that included plastic surgeons, a gynecologist (for patient 1), intensive care specialists, microbiologists, and infectious diseases physicians. Patient 1 was the first case documented in London; the team drew on experience from the US Centers for Disease Control and Prevention, Health Protection Scotland, and the Rare and Imported Pathogens Laboratory at Porton Down, England. Anthrax Immune Globulin Intravenous (Human) (AIGIV) was approved for patient 1, but was not given because she had improved by the time of its arrival. Patient 2 received AIGIV within 24 hours of arrival at the hospital.

### Microbiological Diagnosis

In test results for patients 1 and 2, gram-positive rods were seen on Gram stain from blood culture and *Bacillus anthracis* grew. Gram stain of tissue culture from debridement from patient 2 also showed gram-positive bacilli. Serologic testing and PCR on EDTA blood samples were also positive for both of these patients.

Patient 3 was referred to the vascular surgeons with concern regarding his pseudoaneurysm. He was febrile on admission, and an infectious diseases consultation was requested to evaluate antibiotic drugs in view of skin and tissue cultures which had grown anaerobic organisms. He had injected heroin before onset of illness, although he had not injected drugs for 10 years before this instance. Blood cultures and tissue taken at the time of his pseudoaneurysm repair were negative on culture and by PCR for *B. anthracis*; however, his strongly positive serologic test results were indicative of recent infection.

## Discussion

The death rate early in the 2009–2010 anthrax outbreak in Scotland was ≈50% and dropped to 28% toward the end of the outbreak (*23*). These results are likely to be a reflection of increased vigilance, earlier identification of cases, and an increase in experience in managing the cases. There is speculation that, rather than destroying the contaminated heroin, dealers in Scotland may have “cut,” or diluted, the batch with other supplies; this may have resulted in a reduction in the size of the inoculum. The death rate was also high in the 2012–2013 outbreak, although this outbreak was more limited in extent than the one in 2009–2010.

### Role of Surgery

Regarding soft tissue infections related to heroin-associated anthrax, the role of surgery was initially unclear, and the suggestion was made that disturbing a lesion may result in clinical deterioration, although this hypothesis remains contentious (*20*,*21*,*24*). Based on experiences in Scotland and Health Protection Scotland advice, guidelines from Public Health England (PHE) advocated timely debridement of affected tissue with the aim of reducing the toxin load. Patients 1 and 2 had extensive debridement, but in patient 1 in particular, the extent of involvement and poor demarcation of affected tissue made this difficult. Toward the end of the 2012–2013 outbreak, a more conservative approach was being considered by clinical specialists.

These 3 cases demonstrate the clinical spectrum of anthrax among heroin users from minimal evidence of overt sepsis to soft tissue involvement and multiorgan failure. Several factors are potential contributors to this; these may include delayed manifestation; delays in recognition and subsequently, in initiating therapy; the size and route of entry of the inoculum; comorbidities; and immunologic factors.

### The Tip of the Iceberg

Patient 3 sought treatment mainly for his femoral artery pseudoaneurysm after injecting heroin, although after admission, it was found that he was febrile, had elevated inflammatory markers, and there was evidence of necrosis at debridement. Culture and molecular tests of tissue collected at debridement were negative, possibly caused by early antibiotic drug therapy before that procedure. His manifestation of illness was not initially counted as a case, but this was revised considering the strength of his serologic response which suggested recent infection. This patient’s lack of physical compromise despite active infection is notable. A case report from Glasgow described a patient who had chronic sinuses in the left groin after injecting heroin; there was evidence of local infection and sepsis with *B. anthracis,* but he was was uncompromised (*25*). This raises the possibility that the cases seen in Scotland and England may be the tip of the iceberg, particularly if some patients who are relatively asymptomatic and not overtly septic are treated for soft tissue infections with antibiotic drugs that are effective against anthrax, and those infected persons are not admitted to a hospital.

The seroprevalance of infection in PWIDs who had potentially used heroin from infected batches is unknown and, given the number of heroin users in the United Kingdom, this knowledge would shed further light on our understanding of the immune response to anthrax. This is particularly the case given that some affected individuals may be asymptomatic or minimally symptomatic. Patient 1’s partner injected from the same batch at the same time but showed no evidence of anthrax during follow-up.

Information was disseminated through the press and directly to at risk groups to ensure heightened public awareness; this led several PWIDs to present to the trust concerned regarding possible infection however they proved serologically negative. Community testing of PWIDs may be an important initiative to determine the seroprevalence of anthrax, identify subclinical cases and guide further research into anthrax and immunity.

## Lessons from the 2009–2010 Outbreak

This outbreak reinforces the importance of vigilance in the early identification of emerging infections to ensure rapid identification of cases, containment of an outbreak, and effective management. Early cooperation and dissemination of information to at-risk groups and health care professionals are also vital.

The case-patients in the 2009–2010 outbreak most commonly sought treatment with localized abscesses or inflammatory lesions at the site of injection or skin popping with a breadth of presentation from subclinical to hemorrhagic meningitis and peritonitis (*12*,*20*,*25*–*27*). The novelty of this outbreak is the mode of transmission with endospores injected (in most cases) directly into the blood supply, resulting in novel soft tissue manifestations and severe systemic manifestations. The recurrence of cases in 2012–2013 has reinforced the awareness that vigilance should continue to be exercised in medical practice, looking for links among cases with early reporting and automatic collating of cases so that outbreaks can be identified early.

### Severe Skin and Soft Tissue Infection

The Infectious Diseases Society of America guidelines for skin and soft tissue infections make special mention of the treatment for cutaneous anthrax, given the possibility of anthrax use as a bioterrorism agent (*28*). The recommendations for necrotizing fasciitis include a broad spectrum of antibiotics which are similar to those recommended for the anthrax outbreak; these include ciprofloxacin for suspected anthrax and a combination of a β-lactam antibiotics, ciprofloxacin, and clindamycin for suspected necrotizing fasciitis (*29*). These recommendations are similar to the PHE recommendations for antibiotics to be used in suspected cases of soft tissue anthrax, specifically, ciprofloxacin, clindamycin, and penicillin plus additional cover for necrotizing fasciitis with flucloxacillin and metronidazole (*30*,*31*). The use of antibiotics that have anti-anthrax activity, particularly in severe soft tissue infections in PWIDs, could possibly mask overt cases.

Anthrax immunoglobulin is available on a named patient basis with prior agreement from the US Food and Drug Administration and the Centers for Disease Control and Prevention for patients who fulfill the particular clinical features and who have confirmed laboratory evidence of *B. anthracis* infection in a normally sterile site with epidemiologic evidence of possible infection (*28*). It is derived from plasma of humans vaccinated with BioThrax (adsorbed anthrax vaccine) (Emergent BioSolutions Inc, Bracknell, UK) and contains polyclonal toxin-neutralizing antibodies against protective antigens; it is intended for use as an adjunct to antibiotics to counter the toxin-mediated immune activations (*32*).

### Waste Management

Human-to-human transmission of anthrax has not been reported. However, the clinical waste from patients is potentially hazardous; hence, early recognition and coordination are essential. In 2010, PHE produced updated guidance that highlighted the need to address the threat of potential contamination. This included guidance related to the collection of samples and guidance related to any sharps and contaminated waste that should be incinerated or autoclaved. Anthrax is a Hazard Group 3 pathogen and should be handled in a Biosafety Level 3 facility by using a Class 1 protective safety cabinet (*30*,*31*). All sharps and any waste contaminated by blood or bodily fluids and should be incinerated or autoclaved to interrupt transmission (*31*).

### The Need for Vigilance

Given the novel nature of the clinical manifestation, clinicians may not consider anthrax in the differential diagnosis of severe infections in PWIDs, resulting in undiagnosed cases. Clinical awareness of the associated risk for injection-related infection by rare pathogens in this population is therefore crucial, particularly because the range of clinical manifestation is broad. It is therefore possible that the incidence among PWIDs may be significantly higher for each overt case than that reported for several subclinical cases. The re-emergence of anthrax in 2012–2013, albeit at a smaller scale than in 2009–2010, suggests anthrax-contaminated heroin remains in circulation.

### Other Considerations

For the cases from June 2012, the European Monitoring Centre for Drugs and Drug Addiction in association with the European Centre for Disease Prevention and Control issued a rapid risk assessment highlighting the presence of contaminated heroin in northern Europe and disseminated information to relevant parties (*33*). Of note, there was a delay in diagnosis of 1 fatal case in Germany because of the initial identification of the bacillus as *B. cereus.*

This new form of anthrax associated with PWID reinforces the importance of emerging infections in clinical practice and the potential global impact of such outbreaks. In addition, it highlights how globalization, international travel, and the impact of global conflicts on drug production and supply routes can change the landscapes by which infectious diseases originate and spread. The new cases during 2012 suggest that contaminated heroin remains in circulation.

## Conclusion

Here we describe 3 cases from the 2009–2010 outbreak of heroin-associated anthrax in the United Kingdom. Our interest lies in the way that the anthrax spores were introduced into the body, their novel effects, and breadth of manifestation. Lessons learned from the detection and management of these cases is of renewed interest given the cases seen during June 2012.

Clinicians may not consider anthrax in the differential diagnoses of sepsis and soft tissue infection among PWIDs, leading to an underestimate of the incidence of anthrax in this novel manifestation. The reemergence of anthrax makes a strong argument for serologic surveillance to determine the prevalence of anthrax exposure in PWIDs and to further our understanding of the nature of anthrax immunity and pathogenesis in this cohort. Results of this surveillance would have major implications for health policy in managing an infection for which there is heightened public concern.

Outbreaks among heroin users occur intermittently (*34*), hence vigilance should be exercised for these patients. Ongoing cases in Europe suggest that affected batches of heroin remain in circulation, therefore efficient reporting and noting the association between heroin use and illness are vital in ensuring early awareness of outbreaks.

The possibility that there may be a reservoir of undiagnosed disease, particularly in those with subclinical or mild symptoms, makes a strong case for serologic surveillance among PWIDs seeking treatment for sepsis or soft tissue infections. This will help determine the serologic prevalence in the community and may confirm the possibility that some heroin users possess immunity to anthrax through prior low-level exposure to contaminated drugs. Greater understanding of the nature of anthrax immunity and pathogenesis in this cohort is necessary for the development of health policies targeting this infection for which there is heightened public concern.
